# Commentary on: “Evidence of weak conscious experiences in the exclusion task”

**DOI:** 10.3389/fpsyg.2015.00370

**Published:** 2015-03-31

**Authors:** Gary D. Fisk, Steven J. Haase

**Affiliations:** ^1^Department of Psychology and Sociology, Georgia Southwestern State UniversityAmericus, GA, USA; ^2^Psychology Department, Shippensburg UniversityShippensburg, PA, USA

**Keywords:** unconscious perception, subliminal perception, process dissociation procedure, exclusion task, exhaustiveness

Most studies of unconscious perception aim to demonstrate that the participants are unaware of the prime stimuli (e.g., a measurement of zero sensitivity on prime detection, discrimination, or identification) yet show evidence of perception on another variable (e.g., semantic priming). Dissociation studies of this kind are popular, but the approach has an inherently weak logic. The problem is the need to establish that the awareness measure is exhaustively sensitive to conscious perception (Reingold and Merikle, 1988). The exhaustiveness issue is critical. If exhaustiveness cannot be demonstrated, any findings suggesting unconscious perception can be plausibly attributed to a Type II statistical error (a failure to measure a real effect on the awareness measure) and are open to alternative interpretations.

Sandberg et al. (2014) investigated this exhaustiveness issue by comparing two different methods of measuring awareness in an unconscious perception paradigm. The Perceptual Awareness Scale (PAS) is a direct report of stimulus awareness that is expressed on a four point scale (1: no experience, 2: weak glimpse, 3: an almost clear experience, and 4: A clear experience; Ramsøy and Overgaard, 2004). The PAS was compared to an exclusion task that required the participants to intentionally respond in ways that *did not match* the presented prime stimuli (Debner and Jacoby, 1994; Merikle et al., 1995). Exclusion task results are interpreted as evidence for unconscious perception if the participants match the prime and response at elevated rates (i.e., exclusion failure; an inability to follow instructions presumably due to unconscious perception). Sandberg et al. found significantly elevated prime—response exclusion matches on trials that were rated 1 (“no experience”) or 2 (“weak glimpse”) on the PAS. Exclusion failure at the 2 rating suggested that exclusion failure should not always be interpreted as evidence of unconscious perception because exclusion failure can occur when there is a weak degree of stimulus awareness. The investigators concluded that the PAS is more sensitive (exhaustive) to the presence of conscious perception than the exclusion task. If this is true, then the conclusions of previous studies (e.g., Debner and Jacoby, 1994; Merikle et al., 1995; Smith and Bulman-Fleming, 2004; Matsumoto et al., 2005; Lamy et al., 2008) that interpreted exclusion failure as evidence of unconscious perception may be in doubt because the findings may be partly attributable to conscious perception. Another important implication is that direct measures of awareness (PAS) may be more sensitive than indirect measures (exclusion), which is contrary to the views of some investigators.

We would like to support Sandberg et al.'s conclusions by adding that we have obtained similar findings in an experiment that compared an exclusion task to word discrimination performance (Fisk and Haase, 2007; Experiment 3). The participants observed masked word or nonword stimuli that were presented for 75 ms. Half of the trials contained words; the other half, nonwords. Prime sensitivity was determined after each presentation by asking the participants to indicate the presence of a word on a word-nonword discrimination scale that ranged from 1 (“No word was presented”) to 6 (“Yes, a word was presented”). The second response on each trial was an exclusion task. The results from the word trials showed that exclusion failure did not occur at the low discrimination ratings. In contrast, the exclusion failure rate was above baseline and consistent for trials with ratings three through six. This latter result is similar to Sandberg et al. in that exclusion failure was accompanied by evidence that the prime stimuli were consciously perceived. The word/nonword discrimination sensitivity this experiment was *d_a_* = 0.74, which is well above the zero sensitivity expected for null awareness. When exclusion failure and word-nonword discrimination sensitivity were compared in separate blocks of trials exclusion failure also occurred at display settings with significant prime discrimination sensitivity (58 ms; *d_a_* = 0.35; 75 ms; *d_a_* = 0.82; Experiment 2). Overall, these results are consistent with Sandberg et al. in showing that a direct measure of stimulus perception is more sensitive to the influence of conscious perception than the exclusion task.

Although these studies reached similar conclusions, there were some noteworthy differences in the results. In particular, Sandberg et al. found exclusion failure effects at the lowest level of the PAS (1—“no experience”), but our study (2007, Experiment 3) found no evidence of exclusion failure at the lower levels of the discrimination rating scale (“1” and “2” ratings; “See Figure 3B of Fisk and Haase, 2007”). Methodology differences may partly explain the discrepancies. The key difference is in the task requirements: the PAS emphasizes reporting stimulus awareness or experience, whereas our word discrimination task emphasized confidence that a word was displayed (i.e., distinguishing words from nonwords). There were other methodology differences too, such as the stimulus displays (0–200 ms vs. 75 ms), stimuli presented on the trials (all words vs. 50% words and 50% nonwords) and setting baseline responses (0 ms vs. nonword trials). Aside from the above differences it is not entirely clear why Sandberg et al. found exclusion success (nonmatching, proper performance) at higher ratings of the PAS whereas our results were essentially the opposite (i.e., exclusion failure—matching—at the higher ratings of word discrimination confidence). We, too, have found exclusion success at high ratings in a 2AFC exclusion task (Haase and Fisk, 2001; Fisk and Haase, 2006). Although there are clear differences between these studies, we would like to emphasize again that the general approach and the main conclusions of both studies—that exclusion failure is sometimes accompanied by significant conscious perception of the target stimuli—are essentially the same. Sandberg et al.'s research provides converging evidence that is an important contribution to our understanding of the influence of conscious awareness in the exclusion task paradigm.

Early advocates of using the exclusion task and the Process Dissociation Procedure for studying unconscious perception argued that this approach was advantageous because it circumvented the need to establish null awareness and exhaustive sensitivity (Jacoby and Kelley, 1992; Merikle and Joordens, 1997). In contrast, accumulating evidence from Sandberg et al. and others (Snodgrass, 2002; Fisk and Haase, 2006, 2007, 2013; Bengson and Hutchison, 2007) increasingly suggests that exclusion tasks lack validity for studying unconscious perception. Exclusion failure effects may represent weak conscious perception rather than unconscious perception. Therefore, we feel that investigations of unconscious perception would be better served by using direct ratings of stimulus awareness such as the PAS or other traditional measures, such as detection, identification, and discrimination.

## Conflict of interest statement

The authors declare that the research was conducted in the absence of any commercial or financial relationships that could be construed as a potential conflict of interest.
